# Does Combined Medical and Surgical Treatment Improve Perianal Fistula Outcomes in Patients With Crohn’s Disease? A Systematic Review and Meta-Analysis

**DOI:** 10.1093/ecco-jcc/jjae035

**Published:** 2024-03-16

**Authors:** Moses Fung, Yasamin Farbod, Husain Kankouni, Siddharth Singh, Jeffrey D McCurdy

**Affiliations:** Division of Gastroenterology and Hepatology, Department of Medicine, University of Ottawa, Ottawa, Ontario, Canada; Division of Gastroenterology and Hepatology, Department of Medicine, University of Calgary, Calgary, Alberta, Canada; Department of Medicine, University of Ottawa, Ottawa, Ontario, Canada; Department of Medicine, University of Ottawa, Ottawa, Ontario, Canada; Division of Gastroenterology, Department of Medicine, University of California San Diego, La Jolla, CA, USA; Division of Gastroenterology and Hepatology, Department of Medicine, University of Ottawa, Ottawa, Ontario, Canada; Ottawa Hospital Research Institute, Ottawa, Ontario, USA

**Keywords:** Perianal fistula, combined medical and surgical, anti-TNF

## Abstract

**Background:**

The optimal treatment of perianal fistulizing Crohn’s disease [PFCD] is unknown. We performed a systematic review with meta-analysis to compare combined surgical intervention and anti-tumour necrosis factor [anti-TNF] therapy [combined therapy] vs either therapy alone.

**Methods:**

MEDLINE, EMBASE, and Cochrane databases were searched systematically up to end December 2023. Surgical intervention was defined as an exam under anaesthesia ± setons. We calculated weighted risk ratios [RRs] with 95% confidence intervals [CIs] for our co-primary outcomes: fistula response and healing, defined clinically as a reduction in fistula drainage or number of draining fistulas and fistula closure respectively.

**Results:**

Thirteen studies were analysed: 515 patients treated with combined therapy, 330 patients with surgical intervention, and 406 patients with anti-TNF therapy with follow-up between 10 weeks and 3 years. Fistula response [RR 1.10; 95% CI 0.93–1.30, *p* = 0.28] and healing [RR 1.06; 95% CI 0.86–1.31, *p* = 0.58] was not significantly different when comparing combined therapy with anti-TNF therapy alone. In contrast, combined therapy was associated with significantly higher rates of fistula response [RR 1.25; 95% CI 1.10–1.41, *p* < 0.001] and healing [RR 1.17; 95% CI 1.00–1.36, *p* = 0.05] compared with surgical intervention alone. Our results remained stable when limiting to studies that assessed outcomes within 1 year and studies where <10% of patients underwent fistula closure procedures.

**Conclusion:**

Combined surgery and anti-TNF therapy was not associated with improved PFCD outcomes compared with anti-TNF therapy alone. Due to an inability to control for confounding and small study sizes, future, controlled trials are warranted to confirm these findings.

## 1. Introduction

Perianal fistulizing Crohn’s disease [PFCD] is one of the most challenging phenotypes of Crohn’s disease [CD] to manage. They occur in one in five patients with CD^1,2^ and often follow a relapsing–remitting pattern consisting of frequent episodes of fistula drainage, perianal pain, and abscess formation.^3,4^ As a result, they have a far-reaching impact on the functional, psychosocial, and emotional health of patients living with CD^5–9^ and on healthcare costs.^10^

Despite two seminal clinical trials demonstrating the effectiveness of infliximab, an anti-tumour necrosis factor [anti-TNF] drug, for the induction and maintenance therapy of PFCD,^11,12^ a substantial proportion of patients are unable to achieve sustained fistula remission.^12,13^ As a result, there is considerable interest in strategies to enhance the effectiveness of anti-TNF therapy.^14^ Current guidelines recommend a multidisciplinary approach involving gastroenterologists and surgeons in order to optimize outcomes. This entails an examination under anaesthesia [EUA] with seton[s] to facilitate tract drainage and to prevent abscesses from reforming followed by anti-TNF therapy. Whilst seton insertion is thought to be a crucial component of the initial management of PFCD, its presence prevents complete closure of the fistula tract and may promote aberrant tissue healing through a foreign body reaction.^15–17^ Furthermore, recent studies have raised doubts as to the beneficial impact of setons on long-term PFCD outcomes.^18,19^

Existing guidelines on the management of PFCD are based on observational data and expert opinion. A systematic review by Yassin *et al*. in 2014^20^ demonstrated that combined surgical intervention with anti-TNF therapy resulted in higher rates of fistula healing than either therapy alone. However, their study did not separate surgical intervention from anti-TNF therapy in the cohort who received single modality therapy. Therefore, it is unclear if surgical intervention or anti-TNF therapy is the major driver of fistula healing in patients treated with combined modality therapy. Furthermore, since this publication, there have been a number of additional studies published.^18,19,21–24^

The aim of our current study is to compare the effectiveness of combined surgical intervention and anti-TNF therapy vs either modality alone on PFCD.

## 2. Methods

### 2.1. Study design

We performed a systematic review according to an a priori established protocol and guidance from the Cochrane handbook.^25^ Our study question was designed using the PICO construct: the study population involved patients with PFCD, the intervention was combined surgical and anti-TNF therapy, the comparison was each modality alone, and the outcome was fistula response and remission. Our study was reported according to the Preferred Reporting Items for Systematic Reviews and Meta-Analysis [PRISMA] statement guidelines.^26^

### 2.2. Study eligibility

We included randomized controlled trials, and observational studies [comparative cohort studies] that reported PFCD outcomes and compared combined surgical intervention and anti-TNF therapy with either modality alone. We excluded studies that contained patients with diverting stomas, ileal pouch anal anastomosis, and perianal fistulas related to other aetiologies [e.g. cryptoglandular fistulas]. We also excluded studies that did not stratify outcomes by the type of single modality therapy [surgical intervention or anti-TNF therapy] and studies with insufficient data. We did not restrict studies based on patient age, publication year, publication language, or publication type [full manuscripts or conference proceedings].

### 2.3. Search strategy and study selection

Our search strategy was guided by an experienced information specialist [KF] at the University of Ottawa Health Sciences Library in collaboration with our research team. MEDLINE, EMBASE, and Cochrane Central Register of Controlled Trials [CENTRAL] were searched from inception to December 2023 using the MeSH terms summarized in Supplementary Table 1. To ensure completeness of our search we also reviewed the bibliographies of each included paper, and relevant review articles. Further, we manually searched published abstracts from major international conferences [Digestive Disease Week, European Crohn’s and Colitis Organization, and United European Gastroenterology Week] up to 2023.

Studies were imported into COVIDENCE systemic review software [Veritas Health Innovation], a web-based collaboration software platform designed for study screening and eligibility in systematic reviews. Two reviewers [MF, YF and/or HK] independently evaluated each of the references in duplicate to determine study eligibility. Studies were initially screened by title/abstract with use of pre-established screening questions and those that were identified as potential studies underwent full manuscript retrieval. Disagreements were resolved by consensus [MF and YF, when required by consultation with the senior investigator JM]. Data were extracted in duplicate by two independent reviewers using a pre-designed data extraction template.

### 2.4. Interventions

Combined modality therapy was defined as local surgical intervention by EUA with or without placement of seton[s] and anti-TNF therapy. Anti-TNF therapy was defined as induction and maintenance treatment with infliximab, adalimumab, certolizumab pegol, or golimumab.

### 2.5. Clinical outcomes and quality assessment

Our co-primary outcomes were fistula response and healing. The definitions for fistula response varied between studies and included: minimal fistula symptoms, reduction in the number of draining fistulas, reduction in fistula drainage, or reduction in fistula discomfort. Fistula healing was defined in most studies as the complete closure of fistula tracts clinically and when performed an absence of drainage by the finger compression test. One study included in their definition of fistula healing the lack of complaints by the patient.^27^ In one study, fistula response was the only reported outcome measure and was included in our overall analysis of fistula healing.^28^ Faecal diversion was assessed as a secondary outcome and was defined as diverting ileostomy/colostomy or proctectomy.

### 2.6. Study quality

Study quality was determined using the Good Research for Comparative Effectiveness [GRACE] Checklist, a consensus-based assessment tool for non-interventional studies of comparative effectiveness.^29^ The checklist contains 11 items: six items pertaining to the reporting of data and five items pertaining to the study methods [Supplementary Table 2b]. The data reporting items evaluated the description of treatment exposure, primary outcome recording, objectivity, validity, and equivalency between comparison groups. The methods items evaluated population restrictions, concurrent comparators, confounding/effect-modifying variables, ‘immortal time bias’, and meaningful analyses. Each item was graded as sufficient or insufficient.

### 2.7. Statistical analysis

Statistical analysis was performed using the Cochrane Review Manager [RevMan, v.5.4.1, The Cochrane Collaboration]. Weighted risk ratios [RRs] with 95% confidence intervals [CIs] for fistula outcomes were estimated by fixed effects models, the preferred method for meta-analysis when fewer than five studies are available and when there is minimal heterogeneity between studies.^30^ For analysis involving five or more studies or when significant heterogeneity was present defined as an inconsistency index [*I*^2^] >50%, we used random effects models.^25^ We performed separate analysis comparing combined modality therapy vs either modality alone [anti-TNF therapy or surgical intervention]. To ensure stability of our results, we performed multiple pre-planned sensitivity analysis where we limited our analysis to: [1] studies that used fistula healing as an outcome measure, [2] studies that reported fistula outcomes within 1 year of treatment, and [3] studies where <10% of patients were treated with surgical closure as part of the EUA procedure.

## 3. Results

### 3.1. Study selection and characteristics

**Figure 1.**
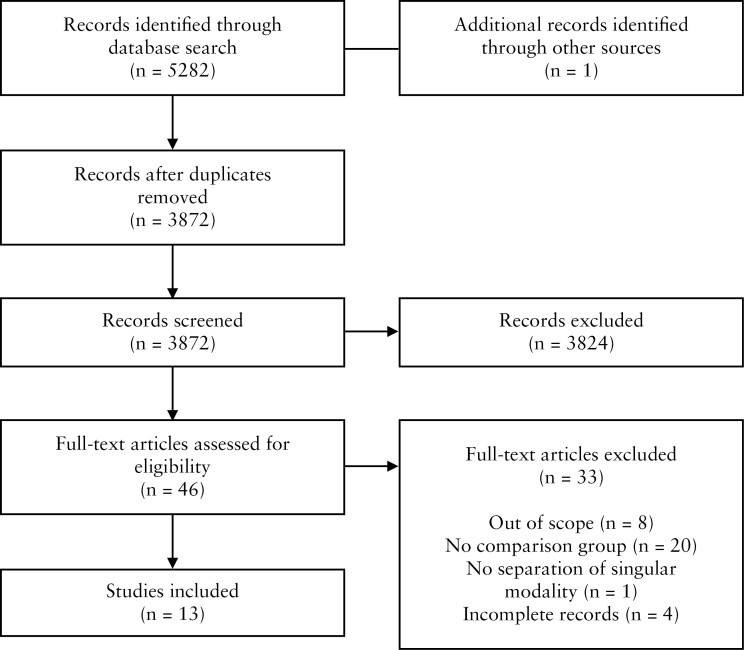
PRIMSA flow diagram of included studies.

We identified 5282 records from our initial search. A total of 46 studies were selected for full-text review and, among these, 33 were excluded: eight that did not assess the study question, 20 that lacked a comparison group, one that did not stratify patients by the type of single modality therapy, and four with incomplete records [Figure 1]. A total of 13 studies were included in our analysis [Table 1]. The majority of studies were single centre [10/13, 77%], retrospective [9/13, 69%], and cohort studies [12/13, 92%]. The studies were conducted in North America [7/13, 54%], Europe [5/13, 39%], or Asia [1/13, 7%]. Seven studies reported on fistula complexity [286/550 complex fistulas, 52%],^27,28,31–35^ five of 13 studies reported on smoking status [194/799 active smoking, 24%],^19,28,32,33,36^ and only one of 13 studies reported on anal stenosis [0/22 with stenosis, 0%].^31^

**Table 1.** Characteristics of included studies

Author	Year	Location	Study type	Males, *n*/*N* [%]	Mean age, years [range]	Mean follow-up, months [range]	Complex fistulas, *n*/*N* [%]	Active luminal disease, *n*/*N* [%]
Regueiro^28^	2003	NA	R SC	16/32 [50.0]	35 [12–58]	36	20/32 [62.5]	32/32 [100]
Ardizzone^31^	2004	Europe	P SC	12/22 [54.5]	38.9 [26.3–51.5]		6/22 [27.2]	22/22 [100]
Van der Hagen^27^	2005	Europe	P SC		34 [22–58]	19 [8–40]	17/17 [100]	17/17 [100]
Gaertner^32^	2007	NA	R SC	105/226 [46.5]	39 [16–83]	30 [6–216]	29/226 [12.8]	128/226 [56.6]
Sciaudone^33^	2010	Europe	P SC	13/35 [37.1]	35 [16–65]	18.8 [8-38]	35/35 [100]	34/35 [97.1]
Uchino^34^	2011	Asia	R SC	43/62 [69.4]	27.5 [16–55]		51/62 [82.3]	
Goldner^38^	2011	NA	R SC	11/15 [73.3]	14 [8–19]			
Cegielny^37^	2012	Europe	R SC					
El-Gazzaz^36^	2012	NA	R SC	82/218 [37.6]	38.8 [26.6–51]	38.4		111/218 [50.9]
Bouguen^35^	2013	Europe	R MC	61/156 [39.1]	30 [13–84]		128/156 [82.1]	156/156 [100]
Schwartz^21^	2015	NA	RCT MC	6/21 [28.6]				
Chan^19^	2022	NA	R SC	100/188 [53.2]	34.7	55.2 [28–153]		85/188 [45.2]
McCurdy^24^	2023	NA	R MC					

NA: North America, R: retrospective cohort study, P: prospective cohort study, RCT: randomized controlled trial, SC: single centre, MC: multi-centre.

### 3.2. Interventions

There were a total of 1251 patients: 515 patients treated by combined surgical intervention and anti-TNF therapy, 330 patients treated by surgical intervention alone, and 406 patients treated by anti-TNF therapy alone. Among patients who underwent an EUA, 575 [69%] patients had seton[s] placed, 173 [21%] patients underwent a fistulotomy, and 92 [11%] patients underwent surgical closure [Table 2]. There were minor differences noted in the types of surgical interventions between the combined modality therapy group and the surgical intervention alone group: setons (82% [422/515] vs 58% [192/330]), fistulotomy (9% [48/515] vs 38% [125/330]), and surgical closure (9% [46/515] vs 14% [46/330]) respectively. The type of anti-TNF therapy included infliximab in 787 [85%] patients, adalimumab in 105 [11%] patients, certolizumab pegol in 21 [2%] patients, and golimumab in one [0.1%] patient.

**Table 2.** Surgical interventions in patients treated with combined modality therapy and surgical intervention alone

Author	Year	Patients receiving surgical intervention	EUA only [*n*]	EUA + seton [*n*]	EUA + seton + fistulotomy [*n*]	EUA + fistulotomy [*n*]	Adv. flap [*n*]	LIFT [*n*]	Glue/plug [*n*]	Other [*n*]
**Combined modality group** ^*^										
Regueiro^28^	2003	9	0	9	0	0	0	0	0	0
Ardizzone^31^	2004	3	0	3	0	0	0	0	0	0
Van der Hagen^27^	2005	10	0	0	0	2	8	0	0	0
Gaertner^32^	2007	79	0	49	0	18	2	0	10	0
Sciaudone^33^	2010	14	0	14	0	0	0	0	0	0
Uchino^34^	2011	26	0	26	0	0	0	0	0	0
Goldner^38^	2011	6	0	0	6	0	0	0	0	0
Cegielny^37^	2012	21	0	21	0	0	0	0	0	0
El-Gazzaz^36^	2012	101	0	53	14	8	15	0	0	11
Bouguen^35^	2013	84	0	84	0	0	0	0	0	0
Schwartz^21^	2015	10	0	10	0	0	0	0	0	0
Chan^19^	2022	66	19	47	0	0	0	0	0	0
McCurdy^24^	2023	86	0	86	0	0	0	0	0	0
**Surgical intervention alone group**										
Van der Hagen^27^	2005	7	0	0	0	4	3	0	0	0
Gaertner^32^	2007	147	0	63	0	74	3	1	6	0
Sciaudone^33^	2010	10	0	10	0	0	0	0	0	0
Uchino^34^	2011	36	0	36	0	0	0	0	0	0
Cegielny^37^	2012	13	0	13	0	0	0	0	0	0
El-Gazzaz^36^	2012	117	0	37	33	14	24	0	0	9

^*^Combined modality: local surgical intervention and anti-TNF therapy, EUA: examination under anaesthesia, Adv: advancement flap, LIFT: ligation of intersphincteric fistula tract.

### 3.3. Outcome measures and timing of outcome assessment

A total of 12 [92%] studies reported fistula healing, four [31%] studies reported fistula response, and three [23%] studies reported both [Table 3]. The timing for outcome assessments varied between 10 weeks and 3 years [10 weeks,^31^ 3 months,^27,28,34^ 6 months,^24,33^ 1 year,^19,21,35,37^ 2.5 years,^32^ 3 years^36,38]^.

**Table 3.** Fistula healing or fistula response outcomes of included studies

Author	Year	Total patients	Outcome measure	Combined modality	Surgical only	Anti-TNF only
Regueiro^28^	2003	32	Fistula response	9/9 [100]		19/23 [82.6]
Ardizzone^31^	2004	22	Fistula healing	3/3 [100]		10/19 [52.6]
Van der Hagen^27^	2005	17	Fistula healing	10/10 [100]	7/7 [100]	
Gaertner^32^	2007	226	Fistula healing	47/79 [59.5]	88/147 [59.9]	
			Fistula response	64/79 [81.0]	126/147 [85.7]	
Sciaudone^33^	2010	35	Fistula healing	9/11 [81.8]	4/7 [57.1]	4/7 [57.1]
			Fistula response	13/14 [92.9]	9/10 [90.0]	10/11 [90.9]
Uchino^34^	2011	62	Fistula healing	22/26 [84.6]	26/36 [72.2]	
Goldner^38^	2011	15	Fistula healing	2/6 [33.3]		4/9 [44.4]
Cegielny^37^	2012	34	Fistula healing	16/21 [76.2]	4/13 [30.8]	
El-Gazzaz^36^	2012	218	Fistula healing	35/101 [34.7]	31/117 [26.5]	
			Fistula response	72/101 [71.3]	42/117 [35.9]	
Bouguen^35^	2013	156	Fistula healing	63/84 [75.0]		45/72 [62.5]
Schwartz^21^	2015	21	Fistula healing	5/10 [50.0]		6/11 [54.5]
Chan^19^	2022	188	Fistula healing	24/66 [36.4]		50/122 [41.0]
McCurdy^24^	2023	225	Fistula healing	38/86 [44.1]		83/139 [59.7]

### 3.4. Combined modality therapy vs anti-TNF therapy alone

**Figure 2.**
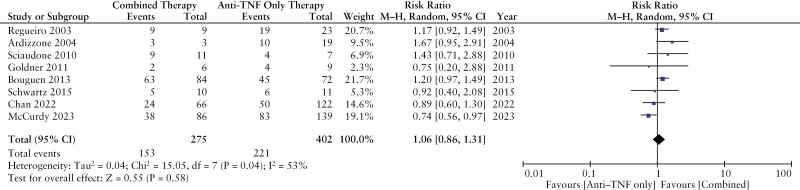
Fistula healing in combined modality therapy vs anti-TNF therapy alone.

Eight studies compared fistula healing between patients treated with combined modality therapy [*n* = 275 patients] vs anti-TNF therapy alone [*n* = 402 patients].^19,21,24,28,31,33,35,38^ Fistula healing occurred in 153 patients [55%] treated with combined modality therapy compared with 221 patients [55%] treated with anti-TNF therapy alone. There was no significant difference in the rates of fistula healing between patients treated with combined modality therapy compared with anti-TNF therapy alone [RR 1.06; 95% CI 0.86–1.31, *p* = 0.58] [Figure 2]. Our results remained stable in a sensitivity analysis where we excluded a study that only reported fistula response [RR 1.04; 95% CI 0.80–1.34, *p* = 0.77]^28^ and when limiting to studies where healing was evaluated within 1 year [RR 1.07; 95% CI 0.86–1.33, *p* = 0.54].^19,21,24,28,31,33,35^ All eight studies had <10% of patients treated with surgical closure, and thus a further sensitivity analysis was not performed.

Two studies compared fistula response between patients treated with combined modality therapy [*n* = 23 patients] vs anti-TNF therapy alone [*n* = 34 patients].^28,33^ Fistula response occurred in 22 patients [96%] treated with combined modality therapy, compared with 29 patients [85%] treated with anti-TNF therapy alone. There was no significant difference in rates of fistula response in patients treated with combined modality therapy compared with anti-TNF therapy alone [RR 1.10; 95% CI 0.93–1.30, *p* = 0.28] [Supplementary Figure 1]. Both of these studies had endpoints within 1 year and had <10% of patients treated with surgical closure, and thus further sensitivity analysis was not performed.

Five studies compared the rates of facal diversion between combined modality therapy [*n* = 83 patients] and anti-TNF therapy alone [*n* = 152 patients]^19,31,33,35,38^; however, only two of these studies delineated which treatment modalities these patients received^19,28^ [Table 4]. Faecal diversion occurred in 11 patients [13%] treated with combined modality therapy and in 25 patients [16%] treated with anti-TNF therapy alone. There was no significant difference in the rates of faecal diversion in patients treated with combined modality therapy compared with anti-TNF therapy alone [RR 0.85; 95% CI 0.46–1.58, *p* = 0.61] [Supplementary Figure 2].

**Table 4.** Faecal diversion outcomes of combined modality therapy vs anti-TNF therapy alone

	Combined modality, *n*/*N* [%]		Anti-TNF only, *n*/*N* [%]		
Author	Diverting ostomy	Proctocolectomy	Diverting ostomy	Proctocolectomy	Total, *n*/*N* [%]
Ardizzone^31^	0/3 [0]	0/3 [0]	1/19	0/19 [0]	1/22 [4.5]
Sciaudone^33^	0/14 [0]^*^		1/11 [9.1]^*^		1/25 [4.0]
Goldner^38^					2/15 [13.3]
Bouguen^35^					19/156 [12.2]
Chan^19^	11/66 [16.7]^*^		23/122 [18.9]^*^		34/188 [18.1]

^*^Study did not specify whether the faecal diversion surgery was a diverting ostomy or proctocolectomy.

### 3.5. Combined modality therapy vs surgical intervention alone

**Figure 3.**
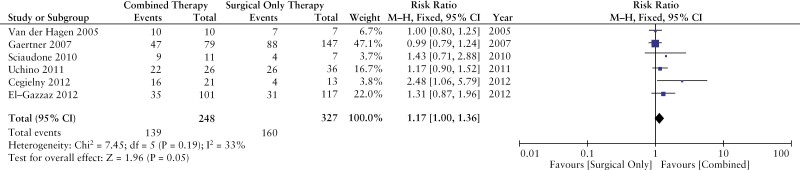
Fistula healing in combined modality therapy vs surgical intervention alone.

Six studies compared fistula healing between patients treated with combined modality therapy [*n* = 248 patients] vs surgical intervention alone [*n* = 327 patients].^27,32–34,36,37^ Fistula healing occurred in 139 patients [56%] treated with combined modality therapy, compared with 160 patients [49%] treated with surgical intervention alone. Fistula healing was significantly more likely in patients treated with combined modality therapy compared with surgical intervention alone [RR 1.17; 95% CI 1.00–1.36, *p* = 0.05] [Figure 3]. Our results remained stable in sensitivity analyses when limiting to studies with endpoints within 1 year [RR 1.33; 95% CI 1.07–1.65, *p* = 0.01]^27,33,34,37^ and when limiting to studies with less than 10% of patients treated with surgical closure [RR 1.42, 95% CI 1.09–1.84, *p* = 0.01].^33,34,37^

Three studies compared fistula response between patients treated with combined modality therapy [*n* = 194 patients] vs surgical intervention alone [*n* = 274 patients].^32,33,36^ Fistula response occurred in 149 patients [77%] treated with combined modality therapy, compared with 177 patients [65%] treated with surgical intervention alone. Fistula response was significantly more likely in patients treated with combined modality therapy compared with surgical intervention alone [RR 1.25; 95% CI 1.10–1.41, *p* = 0.0004] [Supplementary Figure 3]. Further sensitivity analyses were not performed as only one of these three studies reported fistula response outcomes within 1 year^33^ and only one of these three studies had <10% of patients treated with surgical closure.^33^

Four studies compared the rates of faecal diversion [either diverting ostomy or completion proctectomy] between patients treated with combined modality therapy [*n* = 220 patients] vs surgical intervention alone [*n* = 310 patients].^32–34,36^ Faecal diversion occurred in 51 patients [23%] treated with combined modality therapy and in 71 patients [23%] treated with surgical intervention alone [Table 5]. There was no significant difference in the rates of faecal diversion in patients treated with combined modality therapy compared with surgical intervention alone [RR 0.97; 95% CI 0.71–1.33, *p* = 0.86] [Supplementary Figure 4].

**Table 5.** Faecal diversion outcomes of combined modality therapy vs surgical intervention alone

	Combined modality, *n*/*N* [%]		Surgical only, *n*/*N* [%]	
Author	Diverting ostomy	Proctocolectomy	Diverting ostomy	Proctocolectomy
Gaertner^32^	5/79 [6.3]	8/79 [10.1]	9/147 [6.1]	12/147 [8.2]
Sciaudone^33^	0/14 [0]^*^		2/10 [20.0]^*^	
Uchino^34^	4/26 [15.3]	3/26 [11.5]	10/36 [27.8]	9/36 [25.0]
El-Gazzaz^36^	19/101 [18.8]	12/101 [11.9]	15/117 [12.8]	14/117 [12.0]

^*^Study did not specify whether the faecal diversion surgery was a diverting ostomy or proctocolectomy.

### 3.6. Study quality

Study quality ranged from 5 to 8 out of a total of 11 points using the GRACE quality assessment tool [Supplementary Table 2a]. The majority of studies lost points for use of non-validated primary outcomes measures, lack of reporting or control of confounders, potential for ‘immortal time bias’, and lack of subsequent analysis to test key assumptions.

## 4. Discussion

In this systematic review and meta-analysis consisting of 13 studies with 1251 patients, there were no significant differences between combined surgical and anti-TNF therapy compared with anti-TNF therapy alone for PFCD response and healing. In contrast, combined surgical and anti-TNF therapy was more effective in achieving PFCD response and healing compared with surgical intervention alone. Our results remained consistent with multiple sensitivity analysis where we limited the analysis to studies where PFCD outcomes were assessed within 1 year and to studies where <10% of patients underwent surgical closure at the time of EUA. The majority of studies included in our analysis were retrospective, did not control for confounding, and had have low scores on our quality assessment, underscoring the importance of future, randomized controlled trials to determine the true benefit of combined modality therapy for PFCD in patients with CD.

A previous systematic review with meta-analysis consisting of 797 patients found that complete fistula remission occurred more frequently in patients treated with combined modality therapy [180/349 patients; 52%] when compared with either anti-TNF therapy or surgical intervention alone [191/448 patients; 43%]. Similarly, in a retrospective multicentre study from Europe and Israel, medical therapy combined with surgical drainage resulted in higher rates of complete fistula healing [52% vs 42%; *p* = 0.04] and lower rates of repeat surgical intervention [25% vs 59%; *p* = 0.001] compared with either therapy alone.^22^ However, neither of these studies separated anti-TNF therapy from surgical intervention in the single modality cohort. Therefore, the results of our work add to these studies by suggesting that anti-TNF therapy may be the main driver of fistula healing in patients treated with combined modality therapy.

There are number of potential explanations for why combined therapy was not associated with better fistula outcomes compared with anti-TNF therapy alone. It is possible that patients who underwent combined modality therapy had more severe disease and as a result were less likely to achieve fistula response or healing. Alternatively, it is possible that anti-TNF therapy is the major driver of fistula healing, and that local surgical drainage along with seton placement does not provide additional benefit, particularly in the absence of drainable abscesses. It is also unclear what percentage of EUA involved fistula tract curettage, a technique aimed to improve PFCD outcomes by de-epithelializing the tracts.^39^ Finally, it is also possible that any beneficial effect from surgical intervention may have been lost from delayed removal of setons, since setons by design prevent fistula closure. In keeping with this hypothesis, Gaertner *et al*. recently demonstrated that delayed removal of setons was associated with lower rates of fistula closure.^32^

It is important to recognize that few patients in our meta-analysis underwent fistula closure procedures such as advancement flaps or ligation of intersphincteric fistula [LIFT] procedures. PISA-II, a landmark patient preference randomized controlled trial, recently demonstrated that anti-TNF therapy combined with surgical closure improved radiological healing of PFCD compared with anti-TNF therapy.^23^ While this study was not included in our meta-analysis since all patients in both treatment arms underwent an EUA and seton insertion, it demonstrates that surgical closure may be a potential strategy performed at the time of EUA to improve PFCD outcomes. However, it is important to recognize that fistula closure can only be performed in selected patients with optimal outcomes occurring when rectal inflammation and anal strictures are absent.^40^

It was not surprising that combined surgical intervention and anti-TNF therapy was associated with higher rates of fistula healing compared with surgical intervention alone, since, unlike anti-TNF therapy, EUA does not treat the underlying inflammatory mechanisms of CD that are believed to drive fistula activity.^11–13,41^ This was also supported by PISA-I, which demonstrated that EUA with placement of setons alone resulted in higher rates of surgical reintervention [10/15 patients] compared with combined surgical and anti-TNF therapy [6/15 patients].^18^ This study was not included in our analysis since it did not report fistula response or remission as an outcome. There can be considerable variability in the types of interventions performed during an EUA. Fewer patients in the combined therapy group underwent fistulotomy compared to surgical intervention alone [9% vs 38%], suggesting that the combined therapy group may have had more complex disease. Therefore, our results may have underestimated the effectiveness of anti-TNF therapy. In contrast, we do not believe that differences in surgical closure procedures between the cohorts impacted our results, since there were similar rates of surgical closure procedures [9% vs 14%] and our results remained stable in a sensitivity analysis where we limited our analysis to studies where <10% of patients underwent surgical closure.

This is the most comprehensive meta-analysis to date evaluating the impact of combined surgical intervention and anti-TNF therapy compared with either modality alone. The major strengths of our study were our stratified analysis by the type of single modality therapy and the consistency of our results in multiple sensitivity analyses. Our stratified analysis by type of single modality therapy allowed for assessment of the individual effect of surgical intervention and anti-TNF therapy separately in relation to combined modality therapy.

Our study contains a number of important limitations. First, only one study in our analysis was a randomized controlled trial and, among the observational studies, few controlled for confounding such as fistula complexity, concurrent luminal disease, smoking, and anal stenosis. As a result, there may have been bias by indication. Second, the majority of studies in our analysis contained small sample sizes which may impact the precision of our point estimates and limit the generalizability of our findings. Third, standardized protocols for EUA were not used in any of the studies and none reported the proportion of procedures that performed curettage of fistula tracts. Although we performed a sensitivity analysis limiting to studies where <10% of patients underwent surgical closure to help mitigate some of this bias, it remains possible that additional differences in surgical technique existed between study cohorts. Finally, the majority of studies used clinical healing of fistula tracts as their outcome measure without radiological confirmation. However, to date, no widely accepted definition of radiological healing of fistula tracts exists.

Notwithstanding these limitations, there are a number of conclusions that can be drawn from our study. First, surgical therapy should not be used as the sole therapy for complex PFCD. This is consistent with the results of PISA-I and in keeping with societal guidelines.^40,42,43^ Second, exams under anaesthesia ± setons may not be universally required prior to initiating anti-TNF therapy for PFCD. However, in situations where abscesses are present, we still believe that source control, with a dedicated exam under anaesthesia, remains a critical step prior to initiating anti-TNF therapy.

In summary, perianal fistula response and healing in PFCD occurred more frequently in patients treated with combined surgical intervention and anti-TNF therapy compared with surgical intervention alone but not with anti-TNF therapy alone. While these results suggest that combined modality therapy may not be universally required in all patients with PFCD, prospective controlled studies will be necessary to confirm these findings.

## Supplementary Data

Supplementary data are available at *ECCO-JCC* online.

jjae035_suppl_Supplementary_Materials

## Data Availability

The data that support the findings of this study are available on request from the corresponding author [JM].
