# Supplementary material for: Does Combined Medical and Surgical Treatment Improve Perianal Fistula Outcomes in Patients With Crohn’s Disease? A Systematic Review and Meta-Analysis
Source: J Crohns Colitis. 2024 Mar 16;18(8):1261–9. doi: 10.1093/ecco-jcc/jjae035 (PMC11324341; doi:10.1093/ecco-jcc/jjae035)

	Combined Therapy		Anti-TNF Only Therapy			Risk Ratio	
Study or Subgroup	Events	Total	Events	Total	Weight	M-H, Fixed, 95% CI	Year
Ardizzone 2004	0	3	1	19	2.7%	1.67 [0.08, 33.99]	2004
Sciaudone 2010	0	14	1	11	9.1%	0.27 [0.01, 5.97]	2010
Chan 2022	11	66	23	122	88.2%	0.88 [0.46, 1.70]	2022
**Total (95% CI)**		**83**		**152**	**100.0%**	**0.85 [0.46, 1.58]**	
Total events	11		25				

Heterogeneity: $Chi^2 = 0.74$, df = 2 (P = 0.69); $I^2 = 0\%$
Test for overall effect: Z = 0.52 (P = 0.61)

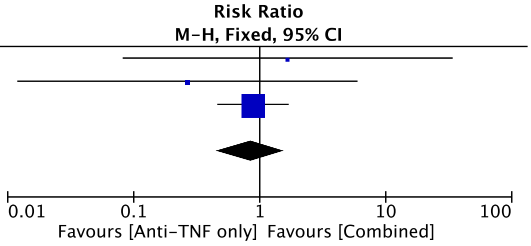

Supplement: jjae035_suppl_Supplementary_Materials [file jjae035_suppl_supplementary_materials.zip › Supplementary Figure_1-4 and Table 1-2/Supplementary Figure 2.pdf]
