# Supplementary material for: Does Combined Medical and Surgical Treatment Improve Perianal Fistula Outcomes in Patients With Crohn’s Disease? A Systematic Review and Meta-Analysis
Source: J Crohns Colitis. 2024 Mar 16;18(8):1261–9. doi: 10.1093/ecco-jcc/jjae035 (PMC11324341; doi:10.1093/ecco-jcc/jjae035)
Supplement: jjae035_suppl_Supplementary_Materials [file jjae035_suppl_supplementary_materials.zip › Supplementary Figure_1-4 and Table 1-2/Supplementary_material.docx]

**FIGURE/TABLE TITLES:**

Supplementary Figure 1: Fistula response in combined modality therapy vs anti-TNF therapy alone

Supplementary Figure 2: Fecal diversion in combined modality therapy vs anti-TNF therapy alone

Supplementary Figure 3: Fistula response in combined modality therapy vs surgical intervention alone

Supplementary Figure 4: Fecal diversion in combined modality therapy vs surgical intervention alone

Supplementary Table 1: MeSH terms used in search

Supplementary Table 2a: GRACE checklist for included studies

Supplementary Table 2b: GRACE checklist definitions

**Supplementary Table 1: MeSH terms used in search**

| **MeSH Terms** |
| --- |
| inflammatory bowel diseases/ OR (inflammatory adj1 bowel adj1 disease*).ti,ab. OR ibd.ti,ab.  OR (crohn* adj1 disease*).ti,ab. OR (perianal adj1 fistula*).ti,ab. OR rectal fistula/ OR fistula-  in-ano.ti,ab. OR (rectal adj1 fistula*).ti,ab. OR (anorect* adj1 fistula*).ti,ab. OR (perianal adj1  disease*).ti,ab. OR (perianal adj1 crohn* adj1 disease*).ti,ab. OR (anorect* adj1 crohn* adj1  disease*).ti,ab. OR (anal adj1 fistula*).ti,ab. OR (anus adj1 fistula*).ti,ab. OR crohn*.ti,ab.]  AND (exam* adj1 under adj1 anes*).ti,ab |

**Supplementary Table 2a: GRACE checklist for included studies***

| **Author** | **Year** | **D1** | **D2** | **D3** | **D4** | **D5** | **D6** | **M1** | **M2** | **M3** | **M4** | **M5** | **Total Score (/11)** |
| --- | --- | --- | --- | --- | --- | --- | --- | --- | --- | --- | --- | --- | --- |
| Regueiro [28] | 2003 | + | + | + | - | + | + | - | + | + | - | - | 7 |
| Ardizzone [31] | 2004 | + | + | + | + | + | + | - | + | + | - | - | 8 |
| Van der Hagen [27] | 2005 | + | + | + | - | + | - | - | + | - | - | - | 5 |
| Gaertner [32] | 2007 | + | + | + | - | + | + | - | + | + | - | - | 7 |
| Sciaudone [33] | 2010 | + | + | + | - | + | + | + | + | + | - | - | 8 |
| Uchino [34] | 2011 | + | + | + | - | + | + | + | + | + | - | - | 8 |
| Goldner [38] | 2011 | + | + | + | - | + | + | - | + | - | - | - | 6 |
| Cegielny [37] | 2012 | + | + | + | - | + | - | - | + | - | - | - | 5 |
| El-Gazzaz [36] | 2012 | + | + | + | - | + | + | - | + | + | - | - | 7 |
| Bouguen [35] | 2013 | + | + | + | - | + | + | - | + | + | - | - | 7 |
| Schwartz [21] | 2015 | + | + | + | - | + | + | - | + | + | - | - | 7 |
| Chan [19] | 2022 | + | + | + | - | + | + | - | + | + | - | - | 7 |
| McCurdy [24] | 2023 | NA | NA | NA | NA | NA | NA | NA | NA | NA | NA | NA | NA |

GRACE: Good Research for Comparative Effectiveness, *definitions for each component are found in Supplementary Table 2b, D: Data, M: Methods, (+) achieving GRACE criteria, (-) not achieving GRACE criteria, NA: not applicable, total score is a composite of 11

**Supplementary Table 2b: GRACE checklist definitions [29]**

| **GRACE Component Item** |
| --- |
| D1: Were treatment and/or important details of treatment exposure adequately recorded for the study purpose in the data source(s)? |
| D2: Were the primary outcomes adequately recorded for the study purpose? |
| D3: Was the primary clinical outcome(s) measured objectively rather than subject to clinical judgment? |
| D4: Were primary outcomes validated, adjudicated, or otherwise known to be valid in a similar population? |
| D5: Was the primary outcome(s) measured or identified in an equivalent manner between the treatment/intervention group and the comparison group? |
| D6: Were important covariates that may be known confounders or effect modifiers available and recorded? |
| M1: Was the study (or analysis) population restricted to new initiators of treatment or those starting a new course of treatment? |
| M2: If 1 or more comparison groups were used, were they concurrent comparators? If not, did the authors justify the use of historical comparison groups? |
| M3: Were important confounding and effect-modifying variables taken into account in the design and/or analysis? |
| M4: Is the classification of exposed and unexposed person-time free of “immortal time bias”? |
| M5: Were any meaningful analyses conducted to test key assumptions on which primary results are based? |

GRACE: Good Research for Comparative Effectiveness, D: Data, M: Methods
