# Supplementary figures and images for: Does Combined Medical and Surgical Treatment Improve Perianal Fistula Outcomes in Patients With Crohn’s Disease? A Systematic Review and Meta-Analysis
Source: J Crohns Colitis. 2024 Mar 16;18(8):1261–9. doi: 10.1093/ecco-jcc/jjae035 (PMC11324341; doi:10.1093/ecco-jcc/jjae035)

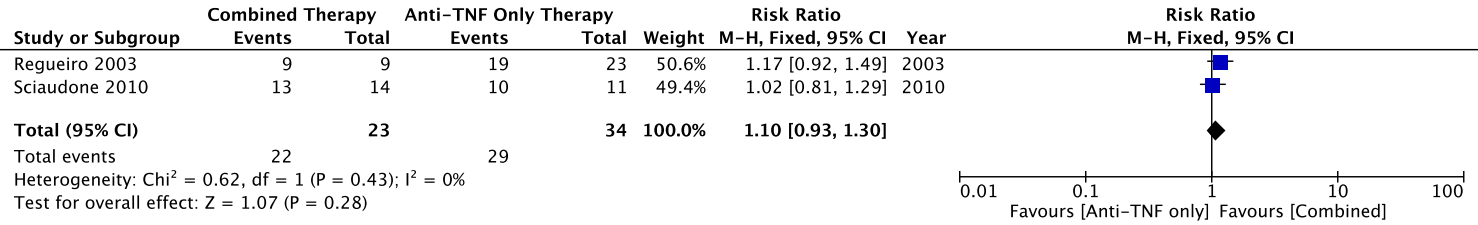

Supplement: jjae035_suppl_Supplementary_Materials [file jjae035_suppl_supplementary_materials.zip › Supplementary Figure_1-4 and Table 1-2/Supplementary Figure 1.pdf]

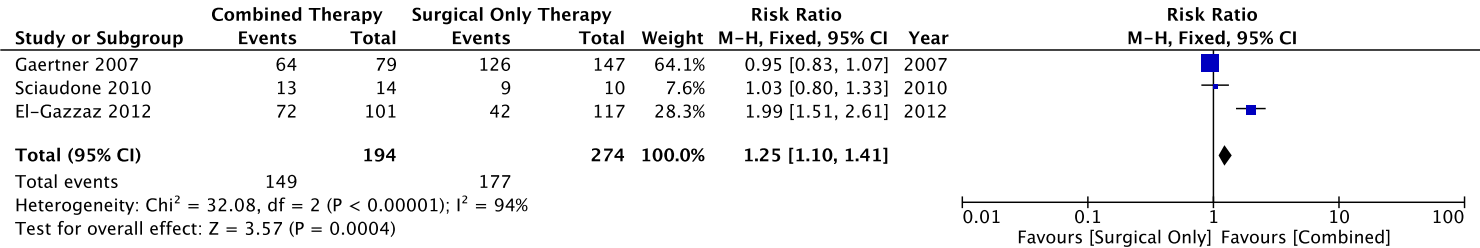

Supplement: jjae035_suppl_Supplementary_Materials [file jjae035_suppl_supplementary_materials.zip › Supplementary Figure_1-4 and Table 1-2/Supplementary Figure 3.pdf]

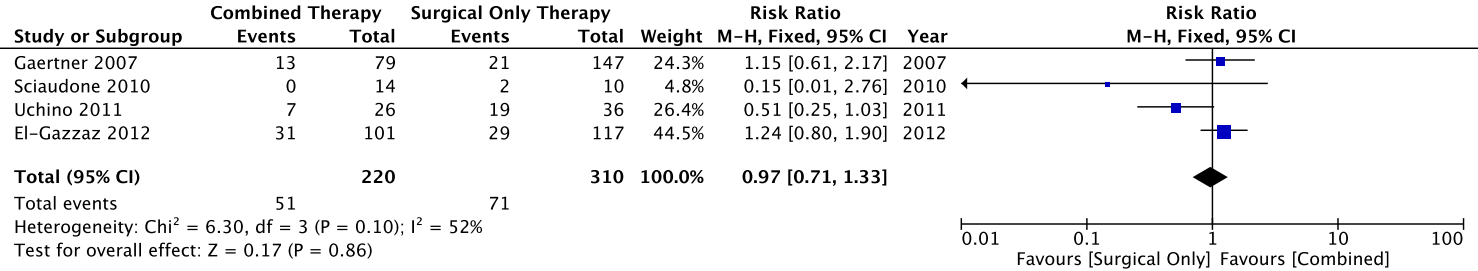

Supplement: jjae035_suppl_Supplementary_Materials [file jjae035_suppl_supplementary_materials.zip › Supplementary Figure_1-4 and Table 1-2/Supplementary Figure 4.pdf]
